# Do human B-1 lymphocytes truly exist?

**DOI:** 10.3389/fimmu.2026.1810144

**Published:** 2026-04-13

**Authors:** Mónica Itzel Martínez-Gutiérrez, Irma Cañedo-Solares, Fernando Gómez-Chávez, Liliana Monserrat Molina-López, Regina Flores-Rabasa, Juan Alejandro Magdaleno-Villanueva, José Israel León-Pedroza, Dolores Correa

**Affiliations:** 1 Plan de Estudios Combinados en Medicina, Facultad de Medicina, Universidad Nacional Autónoma de México, Mexico City, Mexico; 2 Laboratorio de Inmunología Experimental, Subdirección de Medicina Experimental Instituto Nacional de Pediatría, Secretaría de Salud, Mexico City, Mexico; 3 Laboratorio de Enfermedades Osteoarticulares e Inmunológicas, Escuela Nacional de Medicina y Homeopatía, Instituto Politécnico Nacional, Mexico City, Mexico; 4 Centro de Investigación en Ciencias de la Salud, Facultad de Ciencias de la Salud, Universidad Anáhuac México, Huixquilucan, Estado de México, Mexico; 5 Escuela Superior de Medicina, Instituto Politécnico Nacional, Mexico City, Mexico; 6 Hospital General de México “Dr. Eduardo Liceaga”, Secretaría de Salud, Mexico City, Mexico

**Keywords:** B-1 cells, homeostasis, human, innate B cells, natural-antibodies

## Abstract

B-1 cells are a subset of B cells with innate-like properties discovered in the early 1980s in mice. They are important contributors to homeostasis, partly because they secrete natural antibodies with regulatory and scavenging functions. They mainly reside in the pleural and peritoneal cavities, as well as in the spleen and lamina propria of the intestinal mucosa. They differ from conventional (B-2) cells in their ontogeny, phenotype, and functions. In humans, the search for a B-1 cell counterpart began shortly after their discovery in mice, but several difficulties hindered their identification, due to inaccurate phenotypic markers and limited accessibility to the sites where they reside. In this review, we discuss the findings regarding B-1 cells in humans and compare them with murine B-1 cells. In 2011, human B-1–like cells were identified based on functional features and were found to have a CD19^+^ CD20^+^ (or IgM^+^) CD27^+^ CD38^low/int^ CD43^+^ phenotype. This discovery was vigorously debated, but their existence and equivalence to murine B-1 cells has gained acceptance in recent years. These human B-1 cells are present in blood, skin, pleural and peritoneal cavities, the spleen, and several fetal tissues. They exhibit tonic intracellular signaling and spontaneously secrete antibodies that share certain specificities with classical natural antibodies (e.g., against phosphorylcholine or bacterial polysaccharides). However, they also differ from murine B-1 cells in several aspects, including somatic hypermutation and spontaneous secretion of IgG. Several questions remain unresolved regarding their tissue residency, developmental origin during fetal life, and contribution to immune homeostasis. Moreover, evidence addressing their involvement in infectious, inflammatory, and immune-mediated diseases, remains limited and sometimes controversial, although most studies suggest a protective role. In this review, we critically examine these open questions, highlight areas of consensus and debate, and outline future directions necessary to better define the biology and clinical relevance of human B-1 cells.

## Introduction

B lymphocytes are mononuclear cells present in the blood, lymphatic nodes, spleen, mucosa-associated lymphoid tissues and body cavities, such as the pleura and peritoneum (1). Typical classification of B lymphocytes includes three main types: conventional B cells, marginal zone B cells, and B-1 cells. B-1 cells seem to be a unique type of lymphocytes with functional characteristics of both innate and adaptive immune responses. They are differentiated from other B cell types by their origin, phenotype, anatomical location, and the characteristics of the antibodies they produce (1–3).

B-1 cells were first described in mice in 1983 and were initially identified as CD5^+^ B cells (4). Subsequent studies made it clear that a broader phenotype was needed to distinguish these cells from other B cell subtypes, leading to the combination of six surface markers for their correct identification: IgM^hi^ IgD^low^ CD19^hi^ CD23 ^-^ CD43^+^ B220^low^ and, in the case of coelomic cavities, also CD11b^+^ expression (5–8). Murine B-1 cells originate in the para-aortic splanchnopleura and the liver during prenatal development (8). Following differentiation, B-1 cells migrate to their residence sites in the peritoneum, pleural cavity, spleen and intestinal mucosa (2, 6). They are present in small numbers in the adult bone marrow, lymph nodes (7, 9) and peripheral blood, representing ~0.5-1% of total B cells in these sites (10).

Murine B-1 cells are highly dynamic, can migrate to sites of inflammation or infection, differentiate into distinct effector cells (e.g. plasma cells, regulatory B cells, phagocytes, innate response activator cells) and contribute to maintaining homeostasis through the secretion of natural antibodies (NatAbs) or IL-10 (9).

The search for human equivalents of B-1 cells began shortly after their discovery in mice. Numerous studies in the 1980s detected an increased presence of CD5^+^ B cells in patients with chronic leukemia and autoimmune diseases such as rheumatoid arthritis (11–13). In the 1990s, several studies identified CD5^+^ B cells in the peripheral blood of healthy adults, children, and newborns (14). However, the use of CD5 as a distinctive marker for human B-1 cells was discarded, as it is present in a variety of B cells, including naïve, transitional, activated and memory B cells (15–17). This situation raised concerns about the existence of “human B-1 cells” and the features of their presumed NatAbs described in previous works.

In the following years, distinct phenotypes were proposed to identify B-1 cells in humans (6, 18), but several problems hampered their direct comparison with murine B-1 cells, such as the use of inaccurate phenotypic markers, inaccessibility to classical B-1 home sites and controversies surrounding the purity of the isolated cells. However, in 2011, Griffin, et al., proposed an identification approach based on functional characteristics, leading to the phenotype CD19^+^ CD20^+^ CD27^+^ CD43^+^ (15). Although this finding triggered considerable debate, in recent years these cells have gained acceptance (17, 19, 20). Given this controversy, in this review, we analyze current findings on human B-1 cells and compare them with murine ones, to determine their equivalence. We also highlight emerging roles for B-1 cells in health and disease.

To address the heterogeneity and controversy surrounding the identification of human B-1 cells, we applied a structured, three-phase literature screening approach. First, a targeted search was conducted in scientific databases (PubMed, Scopus, and Google Scholar) using the keywords “B-1 cells”, “B-1 lymphocytes”, “innate-like B cells”, “natural autoantibodies”, and “natural antibodies”. This search generated an initial set of key articles, which were compiled into a raw Excel database. Second, duplicate articles were removed, and studies meeting the following inclusion criteria were selected: original and review articles containing human or murine data, with clear methodological descriptions and explicit phenotypic definitions of B-1 cells. Original studies conducted exclusively in mice or already cited in comprehensive reviews were excluded to avoid redundancy. Third, “snowballing” and “reverse snowballing” strategies were applied by searching citations of key articles and their reference lists to identify additional relevant and fundamental studies, respectively. To resolve conflicting findings, the weight of evidence was critically assessed, prioritizing studies with detailed methodological descriptions (e.g., comprehensive selection strategies or phenotype definitions). For original studies, results were directly compared to identify potential sources of heterogeneity and discussed among all authors.

## In search of a phenotype

The search for a normal counterpart to the CD5^+^ B cells in human chronic B cell leukemia resulted in the discovery of murine B-1 cells, initially identified by the expression of CD5 (formerly Ly-1) (21). Following this finding, several studies initiated the identification of CD5^+^ B cells in humans. The frequency of these cells decrease with age, with their highest percentage (75-90% of all B cells) in umbilical cord blood (14). Likewise, these CD5^+^ B cells were identified in patients with autoimmune diseases (mainly rheumatoid arthritis) and chronic leukemia (11–13). Later, it was described that CD5^+^ B cells from umbilical cord blood resembled the autoreactive potential of murine B-1 cells, since they secreted IgM reactive against single-stranded DNA, rheumatoid factor, cardiolipin, vimentin, collagen, chondroitin sulfate and thyroglobulin (14, 22).

The characterization of other human B cell types made it clear that, like murine B-1 cells, sole expression of CD5 could not be used as an exclusive marker as it was present on other circulating B cells, raising questions about the mere existence of human B-1 cell equivalents (15, 17). In 2011 Griffin et al., described B cells with the phenotype CD19^+^ CD20^+^ CD27^+^ CD43^+^ CD70 ^-^ CD69 ^-^ in human cord and adult peripheral blood with three cardinal features of murine B-1 cells: tonic intracellular B cell receptor (BCR) activation in the absence of direct BCR engagement, spontaneous IgM production, and efficient allogeneic T cell stimulation (15). Furthermore, these cells reacted against epitopes canonically recognized by B-1 cells (phosphorylcholine and DNA) (15).

Their discovery and equivalence with murine B-1 cells was vigorously debated due to several controversies (23–26). Early concerns were raised regarding the purity of the isolated B-1 cell populations, as some groups reported substantial T-cell contamination in the form of B:T doublets, which was proposed as an explanation for the co-expression of CD27 and CD43 (24, 25). Furthermore, these studies showed a heterogeneous CD38 expression on CD20^+^ CD27^+^ CD43^+^ cells, reinforcing the idea that the newly proposed B-1 cells were, in fact, activated conventional B cells committed to being plasma cells (plasmablasts) (24, 25). In response, Griffin et al., clarified that they did not observe the aforementioned T cell contamination, as a consequence of sample enrichment with anti-CD19 separation beads (27). They demonstrated the existence of CD20^+^ CD27^+^ CD43^+^ B cells by confocal microscopy and showed that sample dilution and agitation decreased the presence of B:T doublets and thus, the presence of CD3^+^ cells in the CD20^+^ CD27^+^ CD43^+^ gate (27). Furthermore, the analysis of the activation markers revealed that human B-1 cells do not express CD69 nor CD70, which demonstrates they do not represent an intermediate activation state between conventional B cells and plasma cells.

A couple of years later, a broad debate emerged regarding the direct comparison between B-1 cells and conventional plasmablasts (28). In 2013, Covens et al., pointed out that B-1 cells might not represent a distinct B cell lineage but activated cells, as they can differentiate into plasma cells after *in vitro* stimulation. Additionally, several subsets of B cells gain CD43 expression following activation, thereby acquiring a phenotype indistinguishable from that of B-1 cells (23). Furthermore, the same study argued that the putative B-1 cells displayed several features inconsistent with murine B-1 cells, such as gene profile closely related to CD20^-^ plasmablast, spontaneous secretion of different antibody isotypes and the secretion of IgG against a T-dependent antigen (tetanus anatoxine) following vaccination (23). Nevertheless, these results are not inconsistent with the view that human B-1 cells represent a distinct subtype, because it is well known that murine B-1 cells are also capable of differentiating into plasma cells and producing antibodies of different isotypes (9). Moreover, even if other B cells subtypes express CD43 when stimulated, human B-1 cells show a limited differentiation capacity, since only 20% lose CD20 expression and 7% differentiate into plasma cells, while maintaining tonic antibody secretion (23). Regarding T-dependent antigen recognition, Li et al., argued that this could be due to the polyreactive nature of the antibodies secreted by B-1 cells or contamination with other B cell types (29).The same group reanalyzed the microarray data and demonstrated that, across a wide range of distance metrics, gene selection strategies, and numbers of genes analyzed, human B-1 cells consistently cluster closer to memory B cells than to plasmablasts, a result that does not support the interpretation that they might represent activated B cells or preplasmablasts (29).

**Figure 1:**
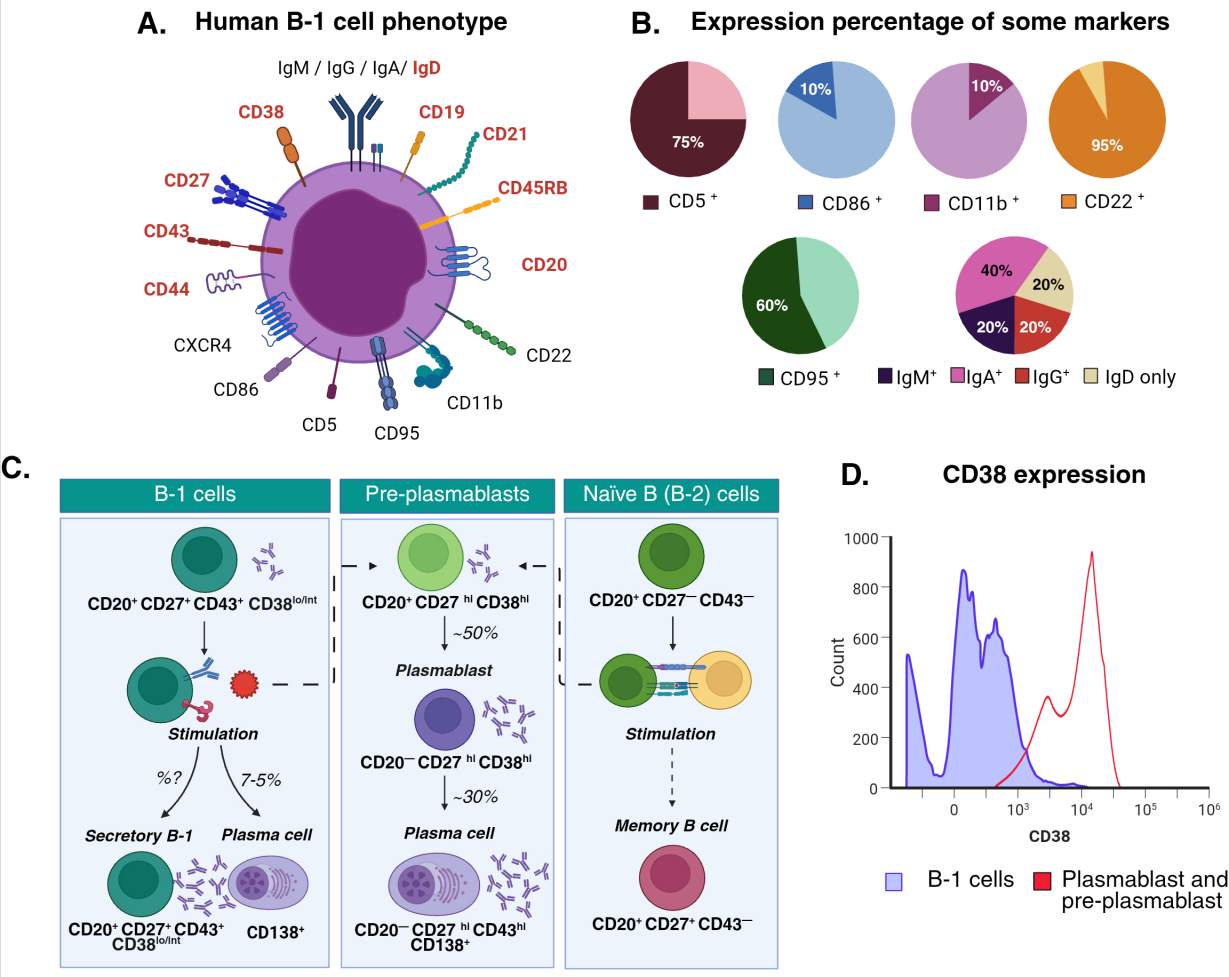
Human B-1 cell phenotype. **(A)** The B-1 cell phenotype has been described in different articles (15, 31). The surface markers in red are present in all B-1 cells. Other markers shown in **(B)** are present in a proportion of these cells, some proposed as markers for cell subtypes. **(C)** The CD38 expression can effectively differentiate B-1 cells from activated B-2 lymphocytes, pre-plasmablasts and plasmablasts (31). After *in vitro* stimulation, approximately 50% of pre-plasmablast lose CD20 expression and ~30% of them gain CD138 expression, indicating their differentiation into plasma cells. In contrast, only 20% of B-1 cells differentiate into CD20 ^-^ plasmablasts (23) and 5% to 7% of them gain CD138 expression after the same stimuli (31). **(D)** Pre-plasmablasts still express CD20 but show a higher expression of CD38 than B-1 cells in steady state (31). Created in BioRender (License: PS29FZIF7B).

All these controversies promoted the use of more rigorous criteria for the isolation and phenotyping of proposed human B-1 cells (16, 30), and underscored the need to compare their functional characteristics with those of other Ig-secreting cells. In 2016, Quach et al., compared B-1 cells with conventional plasmablast (CD20 ^-^ CD27^+^ CD38^hi^), showing their differences in morphology, antibody secretion and differentiation capacity (31). When B-1 cells (CD20^+^ CD27^+^ CD43^+^) are separated by expression, in CD38^low-int^ and CD38^hi^, the latter show characteristics compatible with pre-plasmablasts, since they present greater spontaneous antibody secretion and higher differentiation capacity into conventional plasmablast (CD20^-^ CD27^hi^) and plasma cells (CD138^+^). In contrast, human B-1 cells isolated with the new phenotype (CD19^+^ CD43^+^ CD20^+^ CD27^+^ CD38^low/int^) maintained their spontaneous IgM and IgG secretion (to a lesser extent than CD38^hi^ pre-plasmablasts) and showed overexpression of autoimmunity-related VH sequences (i.e VH4-34). Additionally, these human CD38^low/int^ B-1 cells do not express activation markers (i.e CD69), retain a morphology consistent with non-activated lymphocytes (large, dense nucleus with scanty cytoplasm) and have a limited capacity to differentiate into plasma cells (5% of B-1 cells) (31) (**Figure 1**). Furthermore, these CD38^low/int^ B-1 cells secrete anti-phosphorylcholine (PC) IgM, a classic specificity of B-1-derived NatAbs. Thus, although the origin of CD38^hi^ pre-plasmablasts remains unknown, these results show that human B-1 cells are not activated conventional B lymphocytes or pre-plasmablasts, and represent a distinct cell population within circulating B cells that share several features with murine B-1 cells.

​​In summary, despite the controversies discussed above, current evidence indicates that the ideal phenotype for identifying human B-1 cells is CD20^+^ CD27^+^ CD43^+^ CD38^low/int^. However, the proportion of CD38^hi^ within the total B cell population and the B-1 cell compartment is very low (31) (**Figure 1D**). Therefore, in studies analyzing unstimulated CD20^+^ CD27^+^ CD43^+^ cells (most of which are CD38^low/int^), these cells can reasonably be considered B-1 cells. In some studies, surface IgM is used instead of CD20, which is appropriate since IgM is expressed on the surface of unstimulated B lymphocytes. Therefore, the minimum CD profile we propose to define human B-1 lymphocytes is CD20^+^ (or IgM^+^) CD27^+^ CD43^+^. The specificities and isotypes of the antibodies they synthesize support their classification, as these lymphocytes are natural antibody-producing cells (**Table 1**). Phenotypes that do not conform to these definitions will be termed “B-1-like” cells, since their phenotypic purity cannot be definitively confirmed (see **Supplementary Table 1**).

**Table 1:** Human B-1 cell specificities.

B-1 cell phenotype	Specificity and isotype	Study model	Reference
CD20^+^ CD27^+^ CD43^+^ CD38^low/int^	Anti-PC IgM	Peripheral blood B-1 cells from healthy adults	(15, 31, 32)
	Anti- Neu5GcGM3 IgM	*In vitro* stimulated B-1 cells from healthy 20–88 year-old subjects	(33)
	Anti-dsDNA IgM and IgG. Anti-HEp-2 IgM and IgG.	Cloned antibodies expressing the VH4–34 segment, produced by circulating B-1 cells from 3 healthy adults	(34)
CD19^+^ CD20^+^ CD27^+^ CD43^+^ CD5^+/-^	IgM, IgA and IgG anti- pneumococcal polysaccharides.	Peripheral blood B-1 cells from healthy adults vaccinated against *S. pneumoniae*	(35)
CD20^+^ CD27^+^ CD43^+^	Anti-PS IgG.	Peripheral blood B-1 cells from SLE patients	(36)

dsDNA, double stranded DNA; PC, phosphorylcholine; PS, phosphatidylserine; HEp-2, Hep-2 cell line.

## Locations of human B-1 cells

**Figure 2:**
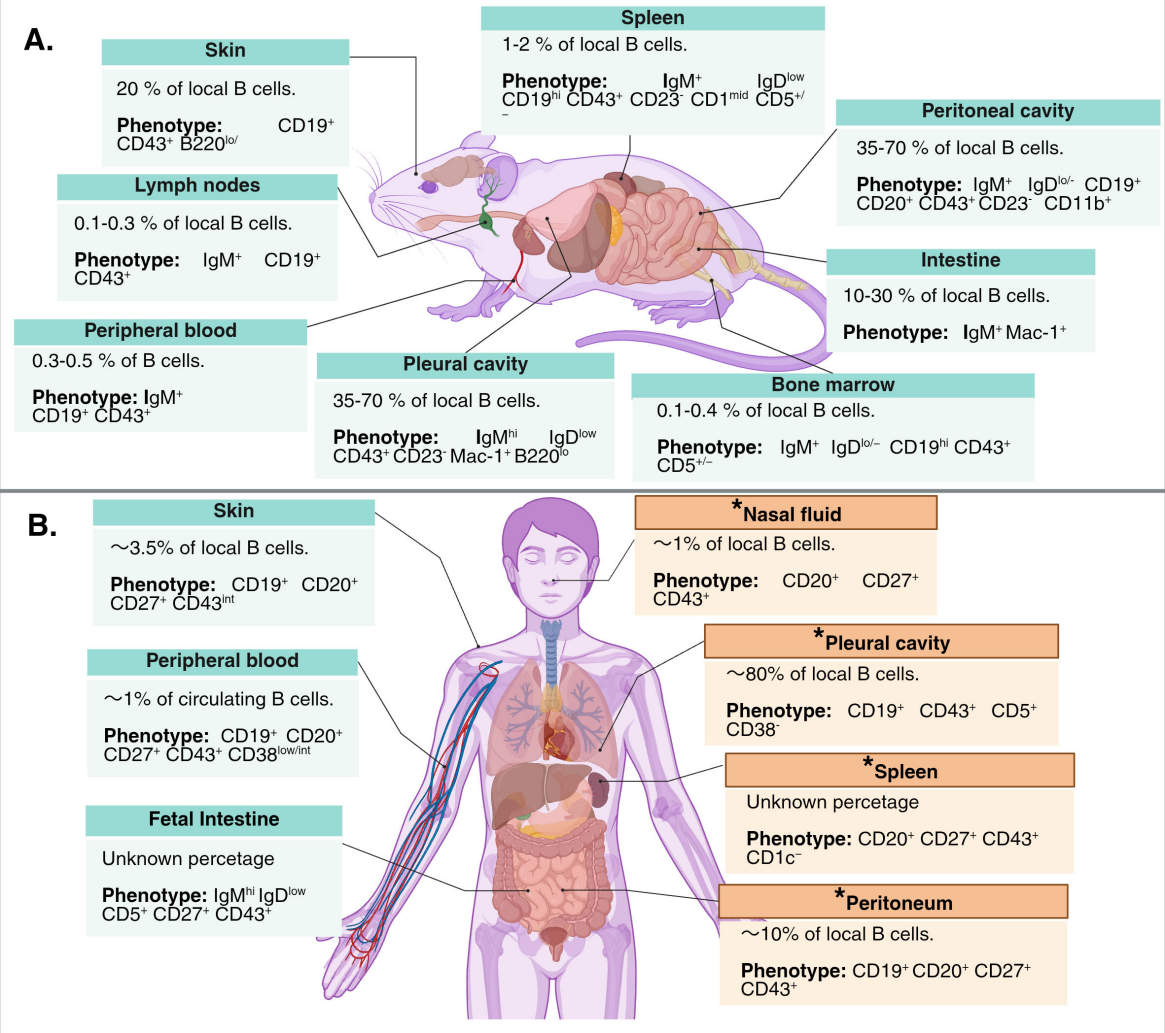
Locations of B-1 cells in mice and humans. **(A)** The main locations in mice are the peritoneal and pleural cavities (5, 6, 39–41), although they can be found in other tissues using a consistent combination of surface markers (6, 21, 42–46). **(B)** In humans, B-1 cells can be identified in different locations, but the lack of use of the complete phenotype (CD20^+^ CD27^+^ CD43^+^ CD38 ^low/int^), opens the possibility of contamination with B-2 pre-plasmablasts, especially in the studies that analyze these cells in relation with NatAbs. Additionally, the majority of the possible resident sites have been described in disease-related conditions (marked with an asterisk), such as kidney transplantation (47), viral infection (48) or plausible systemic inflammation (49); making uncertain the locations of B-1 cells in steady state. Furthermore, their presence in some locations has only been reported in fetal tissues (i.e. fetal intestine) (38). Created in BioRender (license: UQ29FZI2G6).

The classic homing sites of B-1 cells in mice are body cavities and spleen (2, 9). In response to infection, some murine B-1 cells lose CD11b expression and migrate from cavities to the spleen and lymphoid tissues, becoming cytokine- and antibody-secreting cells (8). This migration has not been demonstrated in humans, but some homing sites similar to those observed in mice have been described. Taking the CD27^+^ CD43^+^ CD20^+^ phenotype for human B-1 cells, a wide distribution of these cells has been reported in human tissues. Interestingly, there is more information on the location of B-1 cells in fetuses than after birth: they are present in the liver, bone marrow, yolk sac (YS), spleen, skin, gut and thymus, suggesting a similar origin to those of murine (37, 38) (**Figure 2**). Besides, this highlights their importance in early and not-so-early immune responses.

Peripheral blood B-1 cells (CD20^+^ CD27^+^ CD43^+^ CD38^low/int^) are the most studied due to their accessibility; they represent less than 2% of total circulating B cells in adult and cord blood (15, 31, 37) (**Figure 2**). In both cases human B-1 cells present tonic intracellular activation and spontaneous IgM secretion against PC and ds-DNA.

Their presence in body cavities has been studied since the beginning. An early study in 1997 proposed that “B-1 like” cells (CD19^+^ CD5^+^) accounted for up to 63% of B cells in the adult peritoneum (50). In 2018, Lee et al., reported the presence of B-1 cells (CD20^+^ CD27^+^ CD43^+^ CD1c^-^) cells in the peritoneum of brain-death patients, but did not quantify them (49). B-1 cells defined with the same markers were found to be ≈10% of total B cells in the peritoneal fluid of dialysis patients (47). Their frequency had a moderate correlation with anti- blood group A IgM titers, however, CD38 levels were not analyzed, so it is uncertain whether the antibody titer is related to B-1 cells or represents a mixed response of B-1 cells and CD38^hi^ pre-plasmablasts. Another study confirmed the presence of human B-1 cells (CD20^+^ CD27^+^ CD43^+^) in the omentum of patients undergoing bariatric surgery, where they represent ≈8% of local B cells and have a moderate correlation with anti-PC IgM serum levels (51). Even though this study show human peritoneal B-1 cell frequency is higher than in peripheral blood, the discrepancy with levels reported in murine peritoneum (35–70% of all B cells) (21) could be the result of interspecies differences, migration in response to diseases affecting the peritoneum (47), incomplete phenotypic identification, or a combination of these factors. In any case, the precise data on this localization in humans requires further investigation.

Another controversial location of human B-1 cells is the spleen. Lee et al., reported the presence of B-1 cells (identified as CD20^+^ CD27^+^ CD43^+^ and CD1c−) in spleens of brain-dead organ donors, but did not analyze their proportion within B cells. They reported that IgM and IgG secretion from these cells was enhanced by CD4^+^ CD49^hi^ T cells, complicating interpretation, and did not examine their CD38 expression or some properties of their antibodies to attribute them an NatAb nature (49). Therefore, a quantitative comparison with mouse splenic B-1 cells or other locations in humans is not yet possible.

Evidence for the presence of these cells in mucosal tissues is limited. One study identified B-1 cells (CD19^+^ CD27^+^ CD43^+^) in the nasal lavage fluid of children with bronchiolitis (48), suggesting that the nasal mucosa is another site of B-1 cell residence. However, analysis of healthy children and adults is required to confirm this finding, as it is possible that B-1 cells migrate there in response to an upper respiratory tract infection. On the other hand, using single-cell RNA sequencing, antigen receptor sequencing, and spatial transcriptomics, Suo et al. (38), demonstrated the location of human CD5^+^ B-1 cells (IgD^low^ IgM^+^ CD27^+^ CD43^+^ CD5^+^) in the fetal intestinal epithelium of fetuses up to 17 weeks of age; this is congruent with findings in mice, in which it was demonstrated that some of the IgA (and IgM) secreted into the lumen of the intestinal tract is produced by murine B-1 cells (42, 52).

Other anatomical locations remain understudied. In pleural effusions from patients with bacterial infection, over 80% of B cells have a “B-1 like” phenotype (CD19^+^ CD5^+^ CD43^+^ CD38^-)^ (53). Although characterized without CD27, a proportion of these cells react against PC and express transcription factors related to murine B-1a cells (i.e.TCF1, LEF1) (53), confirming that this population contains true human B-1 cells. In murine skin, mouse B-1 cells (CD19^+^ B220^lo/–^ CD43^+^) represent approximately 20% of total B cells, of which ≈13% produce IL-10 at steady state. In comparison, human B-1 cells (CD20^+^ CD27^+^ CD43^int^) cells in healthy human skin also secrete IL-10 following innate-like stimulation, but represent approximately 3.5% of total B cells (54).

Therefore, these findings demonstrate that while human B-1 cells resemble the conserved functional features of murine B-1 cells, their frequency, tissue distribution, and possibly their influence on the microenvironment differ from those observed in mice. Furthermore, since various anatomical locations (e.g., nasal fluid, spleen, pleural and peritoneal cavities) were studied in patients with inflammatory or infectious conditions (**Figure 2B**, marked with an asterisk), these findings are likely influenced by the clinical context, which complicates the study of B-1 cell residence sites. Direct comparisons with healthy subjects, where possible, are required to define the locations of human B-1 cells under steady-state conditions.

## Human B-1 cells possible subtypes

Several studies have suggested that human B-1 cells can be classified into subtypes based on their functional similarities to murine B-1 cell subtypes. The main surface markers used for this purpose are CD5 and CD11b (**Table 2**). Importantly, none of these studies excluded CD38^hi^ cells (preplasmablasts).

**Table 2:** Main characteristics of human B-1 cell subtypes.

Surface marker	B-1 cell (CD20^+^ CD27^+^ CD43^+^) subtypes		Reference
CD5	CD5^+^ B-1 cells	• Approx. 75% of all B-1 cells. • Mainly produce IgM against pneumococcal polysaccharides after vaccination.	(15, 35, 55)
	CD5^-^ B-1 cells	• Approx. 25% of all B-1 cells. • Production of IgM, IgG and IgA against pneumococcal polysaccharides.	
CD11b	CD11b^+^ B-1 cells	• 8-10% of all B-1 cells. • Spontaneous secretion of IgM and IL-10. • Efficient stimulation of T cells. • TNF-α production suppression by T cells. • Increased in SLE patients.	(56, 57)
	CD11b^-^ B-1 cells	• 89-91% of all B-1 cells. • More spontaneous secretion of IgM than CD11b^+^. • Bare secretion of IL-10 • No influence on TNF-α expression by T cells.	

*SLE, systemic lupus erythematosus.*

### B-1a (CD5^+^) and B-1b (CD5 ^-^) cells

CD5 is a transmembrane glycoprotein and a negative regulator of BCR signaling. It contributes to IL-10 secretion and modulates B cell surveillance and metabolism (58, 59). In mice, B-1 cells are classically divided into two main subtypes, B-1a (CD5^+^) and B-1b (CD5^-^) (3, 60). Murine B-1a cells are the most abundant and the main subset in the spleen and peritoneum, although they are also present in the pleural cavity and bone marrow (10, 39, 43, 61). They are the main source of natural IgM, and their repertoire is restricted, with virtually no N-nucleotide insertions. Furthermore, B-1a cells exhibit high plasticity, giving rise to various effector cells, including innate immune response activators (IRAs) and IL-10-secreting B-1 cells (3). On the other hand, murine B-1b cells have a broader repertoire with frequent N-nucleotide insertions, undergo class switching more readily than B-1a cells, and also act as memory cells after infection (3, 62). Their differences are explained in more detail in (**Supplementary Table 2**).

A relevant scenario for the division of functions between murine B-1a and B-1b cells is pneumococcal infection. B-1a cells are the main producers of natural IgM against PC and against the capsular polysaccharide PPS-3, which protects uninfected mice. However, B-1b cells are the main responders to vaccination and undergo class switching to IgG3. In addition, B-1b cells act as memory B cells, providing long-lasting protection against reinfection (63).

Although CD5 was ruled out as an exclusive phenotypic marker in humans, subsequent studies demonstrated that CD5^+^ and CD5^-^ human B-1 cells (CD20^+^ CD27^+^ CD43^+^) can correspond to murine B-1a and B-1b cells during pneumococcal vaccination. In 2012, Verbinnen et al. (35), showed that 7 days after vaccination with pneumococcal polysaccharides, both types of human B-1 cells produce antibodies, but CD5^-^ B-1 cells were the main producers of polysaccharide-specific IgA, IgG, and IgM. Furthermore, analysis of the B-1 cell compartment in a patient with selective polysaccharide antibody deficiency revealed that 96% of these B-1 cells were CD5^+^, reinforcing the role of CD5^-^ B-1 cells in the T cell-independent type 2 antigen response (35). An independent study confirmed that human B-1 cells respond early to the vaccine, and most CD5^-^ B-1 cells are IgM^-^ on day seven after immunization. However, the predominance of the polysaccharide-specific human B-1 cell subpopulation varies depending on the polysaccharide analyzed (55). These conflicting results show that, while CD5^+^ and CD5^-^ human B-1 cells appear to be functionally distinct, their equivalence to B-1a and B-1b cells needs to be clarified.

### “Orchestrator” (CD11b^+^) and “secretor” (CD11b^-^) B-1 cells

CD11b is a subunit of the integrin Mac-1 and an important surface marker of peritoneal B-1 cells in mice (6). In humans, CD11b identifies two subsets of peripheral B-1 cells, termed “orchestrators” (CD20^+^ CD27^+^ CD43^+^ CD11b^+^) and “secretors”(CD20^+^ CD27^+^ CD43^+^ CD11b**^-^**). CD11b**^-^** B-1 cells specialize in the spontaneous IgM secretion, while CD11b^+^ B-1 cells effectively stimulate T cell proliferation, spontaneously secrete IL-10, and suppress T cell TNF-α secretion, suggesting a similarity between human CD11b^+^ B-1 cells and murine B-1a cells (56, 57). Furthermore, CD11b^+^ cells are increased in patients with SLE, suggesting that they may be involved in the pathophysiology, although it is unclear how (56, 57). None of these studies have linked the expression of this integrin to a migratory state or return to a specific anatomical location, as has been observed in murine B-1 cells in response to influenza infection (19, 64).

There is controversy surrounding these subtypes due to a microarray analysis that showed the expression of T cell-related genes (e.g., CD8α, CD3, CD2, CD7, granzyme A) within CD11b^-^ B-1 cells, as well as the overexpression of myeloid genes (e.g., SIRPα, CD33, CD14, CD68) in the CD11b^+^ B-1 cell subset, leading to the conclusion that CD11b^+^ B-1 cells were in fact monocytes and CD11b^-^ B-1 cells, T-B cell duplets (26). Nevertheless, minimal T cell contamination within CD11b^-^ B-1 cells was confirmed by flow cytometry, and CD11b^+^ B-1 expressed immunoglobulin light chains and surface B cell markers (e.g. CD19, CD79a, CD21) at a level similar to that of naïve and memory B cells, excluding that they are monocytes (65). Thus, these observations need to be confirmed or addressed by other means.

Finally, the co-expression of CD11b and CD5 in B-1 cells has also been studied. Most adult human CD11b^-^ B-1 cells express CD5, and almost all CD11b^-^ B-1 cells from umbilical cord blood are CD5^+^. In contrast, CD11b^+^ B-1 cells exhibit CD5^+^ and CD5^-^ populations (56). Therefore, the functional similarities observed between human CD11b^+^ B-1 cells and murine B-1a cells do not fully correlate with their CD5 expression pattern, highlighting the differences between the two species and the need for further characterization of these subtypes in healthy subjects and in patients with autoimmune diseases.

## Natural antibodies and B-1 cells

NatAbs are classically defined as polyreactive, low-affinity, germline-encoded Ig with a biased repertoire, generated independently of T cells and secreted even in the absence of exogenous antigenic stimulation (26, 66, 67). Splenic and bone marrow murine B-1a cells are the main producers of NatAbs (with a smaller contribution from MBZ cells), and the predominant isotype is IgM, although they can be of the IgG and IgA classes (43, 66). A well-known characteristic of NatAbs is their polyreactivity, attributed to their specificity for phylogenetically conserved epitopes (66, 68, 69) such as DNA (70), glycan epitopes (69), and PC, which is present in Gram-positive bacteria, oxidized lipids, and apoptotic cells (60). Some of these antibodies are also autoreactive and perform functions that aid in antibody clearance, such as complement activation and opsonization of senescent and apoptotic cells (68). Furthermore, NatAbs act as a first line of defense, as they are secreted spontaneously and arise early in life before antigen encounter (69).

In humans, NatAbs were described long ago, as Karl Landsteiner demonstrated that individuals with type O blood have antibodies against blood types A and B without immunization. These antibodies were subsequently shown to cross-react with some sugars in the gut microbiota (67). However, comparison with murine NatAbs and their NatAb-secreting cells faces several challenges. First, in humans, antigenic exposure cannot be controlled, making it impossible to study antibodies in a pre-immune state. Second, most murine NatAbs lack N additions and somatic hypermutation (SHM), but these characteristics are not definitive; that is, murine B-1 cell-derived NatAbs progressively accumulate N additions, SHM, and class switching with age (69, 71). This contrasts with human B cells, which exhibit SHM and class switching even during fetal development (69, 72). Finally, human B-cell populations are not fully represented due to inconsistent use of surface markers, which has limited the study of NatAb-secreting cells and their secreted Ig chemical characteristics (i.e., low affinity and polyreactivity) (73).

In light of the above, it is necessary to redefine NatAb for humans. Here we incorporate criteria from previous analyses to define human NatAb as those present in an individual without known antigenic exposure, or as Ig directed against phylogenetically conserved molecules (66, 68, 69). This definition includes self-reactive Ig, provided they are present in healthy individuals and have the potential to perform protective or homeostatic functions. According to these criteria, humans produce NatAbs that target conserved antigens, including carbohydrates (74, 75), disulfide isomerase (76), and PC. Anti-PC antibodies are of particular interest because they are present from birth to adulthood (77, 78), and their serum levels correlate with cardiovascular protection (78–82). In addition, healthy individuals have NatAbs against autoantigens without known prior exposure, such as human amyloid peptide (83, 84), tumor-associated antigens (33, 85), mutant huntingtin (86), and α-synuclein (87). Furthermore, several studies have shown that natural autoantibodies of different isotypes are ubiquitous in human sera from the neonatal period and react with both intracellular and extracellular compounds (74, 85, 88–90). Analysis of autoreactive IgM suggests that humans have an inherent repertoire of auto-NatAbs at birth, which is subsequently modified by exposure to antigens throughout life (85, 88).

In this regard, human B-1 cells produce NatAb, as their antibodies target conserved molecules such as PC, autoantigens (i.e., DNA), and antigens without known prior exposure, such as tumor antigens. Their reactivity against PC was first detected through their ability to bind to PC-BSA (15), and subsequent studies confirmed that B-1 cells spontaneously produce anti-PC IgM (31, 32). Their autoreactivity was initially confirmed by their ability to bind to DYEWS (a DNA mimetope) and their enrichment in variable region genes associated with autoimmunity (i.e., VH-4-34) (15, 31). Recently, the secretion of anti-DNA IgM and IgG was confirmed (34). Furthermore, B-1 cells show a moderate positive correlation with serum levels of anti-blood group A IgM, although the production of this antibody by different B cell subgroups was not directly compared (47). Regarding NatAbs against antigens of unknown exposure, B-1 cells are the main source of anti-Neu5GcGM3 IgM, a ganglioside overexpressed in several tumors but absent in normal human cells (33, 91). Notably, anti-Neu5GcGM3 antibodies from healthy humans can recognize and eliminate tumor cells expressing this ganglioside, suggesting that B-1 cells play a role in tumor surveillance (92).

Paradoxically, human B-1 cells may be involved in the generation of pathological antibodies. Recently, In 2023, Ma et al., demonstrated that human B-1 cells (CD20^+^ CD27^+^ CD43^+^) are the main producers of anti-phosphatidylserine (PS) IgG in patients with SLE, which is related to disease severity (36); in fact, they worsened kidney damage when transferred to a mouse model of SLE. These human B-1 cells are present in kidney biopsies from patients with class IV lupus nephritis, and their presence correlates with proteinuria levels (36). The fact that these anti-PS antibodies are of the IgG class raises the question of whether they are B-1-derived antibodies or secreted by CD38^hi^ preplasmablasts, which were not eliminated in their study.

Therefore, antibodies produced by B-1 cells can be considered NatAbs, as they target classic murine NatAb antigens, are present in healthy individuals without prior exposure, and can perform maintenance functions. However, other key characteristics, such as low affinity and polyreactivity (67, 69), have not been evaluated in most studies. Notably, human B-1 cells are not the only source of NatAbs, but also MZB cells and even naïve B-2 cells (32, 34, 93, 94). Therefore, further research is needed to describe the composition of the human NatAb-secreting cell pool and the contribution of each B lymphocyte type.

## Age related changes in B-1 cells

### Prenatal and neonatal B-1 cells

Since the 1980s, it has been suggested that murine B-1 cells are derived from committed hematopoietic stem cells (HSCs) in the fetal liver, the newborn spleen, and the bone marrow (95). Subsequent studies demonstrated earlier presence of murine B-1 cell progenitors, as non-HSC precursors in the YS and para-aortic splanchnopleura (P-Sp) (2, 96).

**Figure 3:**
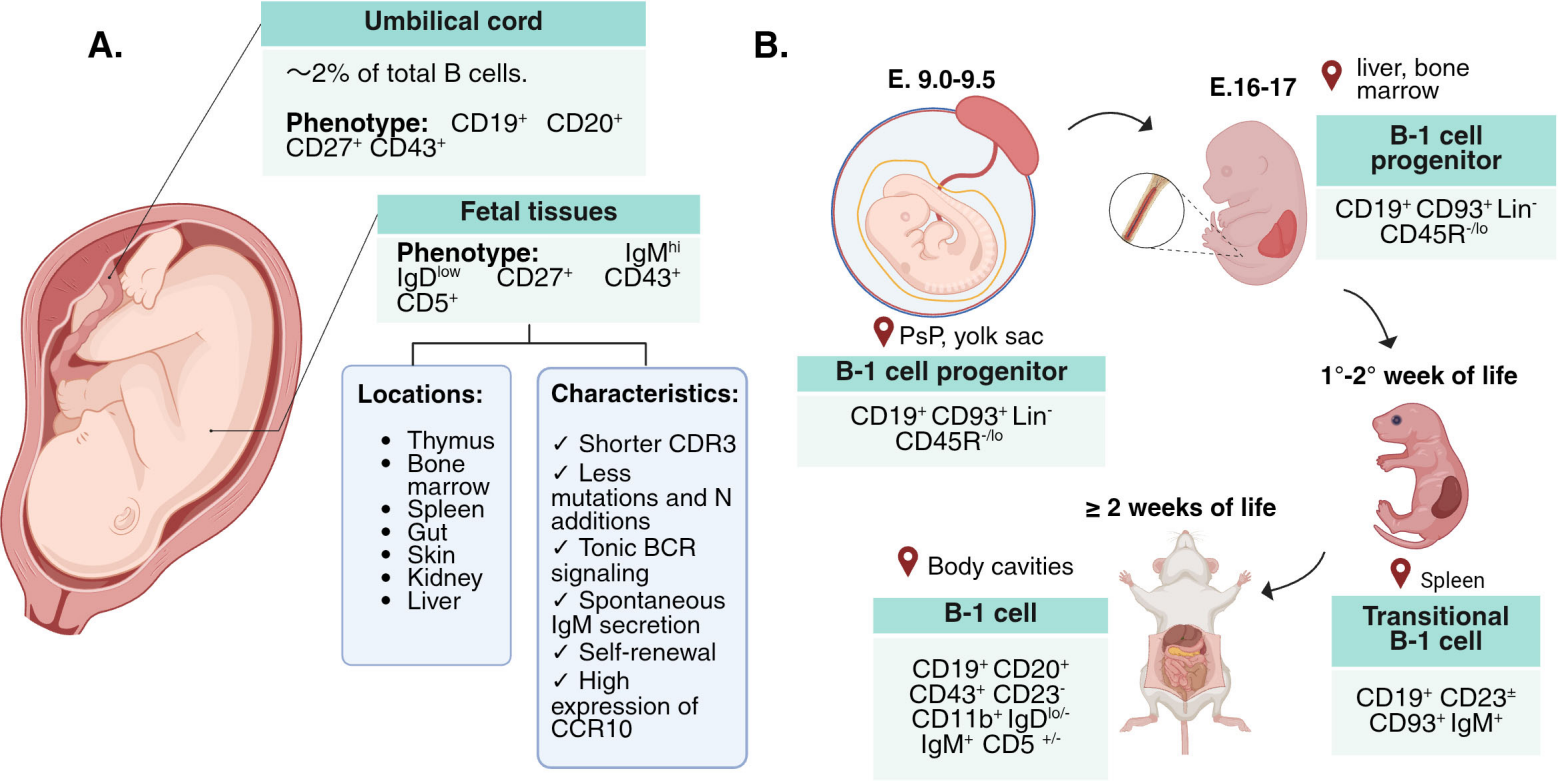
Location of prenatal human and murine B-1 cells. **(A)** Human B-1 cells have been identified in umbilical cord blood and fetal tissues, and they exhibit functional characteristics typical of murine B-1 cells, such as spontaneous antibody secretion, high expression of CD27 and CD43, and features indicative of tonic BCR signaling and self-renewal capacity. **(B)** The exact pathways and mechanisms of murine B-1 cell migration are not fully understood. Transitional B-1 cells may migrate from the spleen to body cavities via the omentum, guided by CXCL13 in mice (19, 39). Such migration has not been studied in human B-1 cells, although the CCL28 has been proposed as the main chemokine involved in human B-1 cell homing in the fetus (38). Created in BioRender (License: WX29FZIPL9).

However, the ontogeny of murine B-1 cells remains a subject of debate, as there is evidence for two different developmental models (more extensive reviews in references (9) and (19). The “selection model” postulates that BCR-biased antigen recognition instructs a common B-cell progenitor to develop into either a murine B-1 or B-2 cell (97). On the other hand, the “lineage model” proposes that B-1 cells arise from a committed progenitor identified by the Lin^−^ CD93^+^ CD19^+^ B220^lo/neg^ phenotype in three waves, each with different potential to generate B-1 cell precursors. The first wave begins in murine embryonic tissues such as the YS and P-Sp, where non-HSC cells have the greatest capacity to generate B-1 progenitors. During the second wave, which begins in mid-gestation in the murine fetal liver and bone marrow, HSCs generate B-1 progenitors from which mature B-1 cells develop. The third wave occurs in the neonatal bone marrow, but its efficiency is lower compared to the second wave, as it primarily produces B-2 cells (2, 97). Although it was recently demonstrated that prenatal generation is not the main source of peritoneal B-1a cells in the adult mouse, the extent to which other waves contribute to the reservoirs of adult B-1a and B-1b cells in different niches is unknown (98). Either way, murine B-1 cell precursors give rise to transitional B-1 cells, present in the spleen during the first two postnatal weeks, and are progressively replaced by transitional B-2 cells that eventually predominate in the adult mouse (97). At the same time, B-1 cells begin to accumulate in the murine peritoneum, suggesting that transitional cells migrate from the spleen to the body cavities, where they eventually mature and acquire the B-1a and B-1b phenotypes (2, 19) (**Figure 3B**).

Although the life cycle of human B-1 cells is much less understood, several findings have shed light on our understanding of their prenatal origins. Recently, prenatal B-1 cells (IgM^hi^ IgD^low^ CD27^+^ CD43^+^ CD5^+^) have been described in various fetal tissues, including the liver, the bone the marrow, the spleen, and the skin, even at early stages of development (i.e., seven weeks of gestation) (38) (**Figure 3A**). These fetal B-1 cells exhibit features similar to murine B-1 cells, such as higher IGHM expression compared to conventional B cells and high activity of transcription factors in the TNF-α and NF-κB signaling pathways, suggesting tonic BCR signaling. Furthermore, they possess shorter CDR3 sequences and fewer N/P additions in both light and heavy chains (38). Analysis of V(D)J usage revealed biased expression of certain gene families; however, unlike adult B-1 cells, the VH-4–34 segment was not found among those with a significant difference (34, 38). CCR10 may play a role in prenatal human B-1 cell migration, as its ligand, CCL28, is expressed in various tissues such as the bone marrow, the stroma, the intestinal epithelium, and the skin (38). This prenatal human B-1 cell pool is enriched in early fetal life and declines during the second trimester, except in the thymus (37, 38). This finding is consistent with a previous study demonstrating that the frequency of fetal liver B-1 cells (CD20^+^ CD27^+^ CD43^+^) declines throughout gestation, falling from ~6% of total B cells at weeks 10.5–11 to ~2.1% of total B cells by weeks 20-21 (37). After birth, human B-1 cells account for only ~2% of total cord blood B cells. In contrast, the frequency of naïve B-2 (CD20^+^ CD27^-^ CD43^-^) cells follows an opposite trend, comprising 87% of total B cells in umbilical cord at 20–21 weeks of gestation (37).

Whether the origin of human fetal B-1 cells is better explained by the selection or the lineage model, remains unknown. In NSG mice, Quach et al. (99), found that grafted CD34^+^ Lin- CD38^low/-^ human HSCs give rise to two B cell populations with the B-1 and B-2 phenotypes in the spleen, bone marrow, and peritoneal cavity of mice. Interestingly, the grafted B-1 cells displayed a BCR repertoire similar to that of human umbilical cord B-1 cells (CD20^+^ CD27^+^ CD43^+^). Moreover, transplantation of mobilized HSC into patients with hematologic malignancies, result in the reconstitution of conventional B and B-1 cells (CD20^+^ CD27^+^ CD43^+^ CD38^lo/int^) to levels equivalent to those reported for healthy individuals (1-2% of total circulating B cells) (99), which is in line with other transplantation study (100). These results suggest that human B-1 cells share a common HSC progenitor with conventional B cells. However, human B-1 cell progenitors have not been searched before the sixth week after conception, when the first fully functional HSCs can be detected (101). Therefore, it is still unknown whether human B-1 cells originate from a committed non-HSC progenitor, as proposed by the lineage model in mice.

Regarding their functional characteristics, human newborn B-1 cells are known to be very similar to adult B-1 cells. They exhibit tonic intracellular BCR signaling, spontaneous IgM secretion, and binding capacity to PC and double-stranded DNA mimotope (15). Interestingly, newborn B-1 and conventional B cells express high and comparable levels of CD38 (37). This phenomenon could reflect an accelerated response to stimulation, as previously demonstrated in conventional B cells from umbilical cord blood (102).

### Adult and elder B-1 cells

The proportion of human B-1 cells (CD20^+^CD27^+^CD43^+^CD38^low/int^) within the peripheral blood B cell population decreases and plateaus at 45 years of age, remaining stable thereafter (103). This contrasts with murine B-1 cells, whose numbers remain virtually unchanged with age (104, 105). However, aged human B-1 cells exhibit functional changes such as decreased spontaneous IgM secretion, likely mediated by downregulation of the Ig secretion-related proteins XBP1 and Blimp1, and increased PAX5 expression, similar to non-secretory B cells (103). Furthermore, their repertoire diversity is reduced, and the frequency of B-1 cell clones expressing the VH3–33 gene segment, known to produce antibodies against apoptotic cells and vaccines, is lower in older individuals. Conversely, the genetic segments VH3–23 and VH4-34, associated with autoimmune diseases and hematopoietic neoplasms, are more frequent (103).

These findings show that the beneficial functions of B-1 cells deteriorate with aging, a phenomenon also observed in other human B lymphocytes and murine B-1 cells (105). However, a key difference between human and murine B-1 cells lies in the N-nucleotide insertions and Ig mutations that occur throughout their lifespan. In humans, although fetal B-1 cells show a lower mutation rate in their Ig chains than other B cells, neonatal and adult B-1 cells exhibit levels of N-nucleotide insertions and total Ig mutations similar to those of naïve conventional cells (18, 31, 106). This aligns with the progressive diversification of B cell repertoire previously observed in human fetal development (72). In contrast, most newborn murine B-1 cells possess few or no N-additions and progressively accumulate these mutations with age. This phenomenon is attributed to their fetal origin, when the terminal deoxynucleotidyl transferase (TdT) is not expressed in mice (71, 95).

These differences likely stem from the distinct timing of TdT expression in both species, since humans express TdT since week 8 of gestation (107) and its levels increase since then (108). Furthermore, this relatively high N-addition and Ig mutation rate in human B-1 cells could have an impact in the NatAb mediated protection, since the loss of germline structure in murine B-1a cell-derived natural IgM, leads to a reduced protection against pneumococcal infection (109, 110).

### B-1 cells in different diseases

Although several studies have analyzed “B-1-like” cells in human diseases, most of them used a non-specific phenotype that led to the study of different subtypes of activated B cells, rather than a single cell population (summarized in **Supplementary Table 3**). However, the increasing adoption of the CD20^+^ CD27^+^ CD43^+^ phenotype for human B-1 cells has led to a surge of new research focused on its alterations in various diseases (**Table 3**).

**Table 3:** Studies of B-1 cells in different human diseases.

Category	Condition	Phenotype	Findings	Reference
Infectious diseases	Periodontitis	CD20^+^CD43^+^CD27^+^ CD69^-^ CD11b^+^	• Circulating CD11b^+^ B-1 cells are significantly reduced in patients with severe periodontitis.	(111)
	Bronchiolitis	CD19^+^CD27^+^ CD43^+^ CD70^-^ CD5^+/-^	• B-1 cells were found in nasal lavage fluid from children with bronchiolitis. Their frequency is similar to that described in peripheral blood. The specific effect of bronchiolitis on the B-1 cell pool remains unknown	(48)
Inflammatory and autoimmune diseases	Chagasic Cardiomyopathy	CD19^+^CD20^+^ CD27^+^CD43^+^ CD11b^+^	• The *T. cruzi* PRO fraction induces IL-10 and TNF-α secretion in B-1 cells from indeterminate-phase patients. • CD11b^+^ B-1 cells frequency in blood positively correlates with cardiac function.	(112)
	Dilated Cardiomyopathy (DCM)	CD3^−^CD19^+^ CD20^+^ CD27^+^ CD43^+^ CD38^lo/int^	• Significantly decreased in DCM patients positive for anti-β1−AR antibodies. • Negatively correlated with NT−proBNP levels and positively correlated with the LVEF in patients with DCM.	(113)
	Relapsing– remitting multiple sclerosis	CD20^+^CD27^+^ CD43^+^	• Newly diagnosed patients show a significant reduction in circulating B1 cell frequency. • B-1 cell numbers were inversely correlated with the time elapsed since the last acute episode.	(114)
	Systemic lupus erythematosus (SLE)	CD20^+^CD27^+^ CD43^+^CD70^-^	• CD11b^+^ B1 cells are increased in SLE patients, as well as their expression of CD86, consistent with their capacity to stimulate T cell proliferation. • Are the main source of the pathogenic anti-PS IgG. • The levels of renal-infiltration are positively correlated with proteinuria severity.	(36, 56)
	Bowel Disease	CD20^+^CD27^+^ CD43^+^	• Untreated patients have less circulating B-1 cells than adalimumab/infliximab treated patients and controls.	(115)
Immunodeficiencies	Combined variable immunodeficiency	CD20^+^CD27^+^ CD43 ^lo–int^	• The percentage of B-1 and memory B cells is reduced by more than 50% compared to controls.	(116)
Cancer	Cancer surveillance	CD19^+^CD20^+^ CD27^+^ CD43^+^ CD38^lo/int^	• B-1 cells are the main producers of IgM against the tumor antigen Neu5GcGM3. • IgM Abs eliminate tumor cells through complement-dependent and independent mechanisms.	(33, 92)
Transplants	Antibody-mediated rejection in ABO-incompatible transplants.	B1: CD20^+^CD27^+^ CD43^+^CD1c^−^ MZ−B1: CD20^+^CD27^+^ CD43^+^ CD1c^+^	• In ABO-incompatible kidney transplant patients, B cells reactive to group A antigen are enriched in B-1 and MZ-B-1 types. • Blood levels of B-1 cells have a moderate correlation with serum levels of anti-group A IgM and IgG.	(47)

Abs, antibodies; NT−proBNP, N-terminal pro-brain natriuretic peptide; LVEF, Left ventricular ejection function; β1−AR, β1-adrenergic receptor; PS, phosphatidylserine; PRO, protein-enriched fraction derived from T. cruzi.

It is important to clarify that, in most of the studies presented below, human B-1 cells are identified as CD20^+^ CD43^+^ CD27^+^, which raises questions about the extent of contamination with pre-plasmablasts (i.e., CD38^hi^) of B-1 or B-2 origin.

Infectious diseases

It is well known that murine B-1 cells play an important protective role in various infections (8). In humans, recent studies have explored changes in B-1 cells during two infectious diseases, periodontitis and bronchiolitis, and during pneumococcal vaccination.

Periodontitis is a chronic inflammatory disease that leads to the destruction of the structures that support the teeth (117). B cells appear to contribute to both anti-inflammatory and pro-inflammatory events in periodontitis. Furthermore, mice with B cell depletion show a reduction in periodontitis-induced alveolar bone loss, highlighting their potential pathological role (106). Demoersman et al. (111), studied the distribution of B cells in the peripheral blood of 15 patients with severe periodontitis and found that these patients had higher levels of class-switched memory B cells and reduced levels of B-1 cells (CD20^+^ CD27^+^ CD43^+^ CD69 ^-^), especially the CD11b^+^ subtype. Other authors have described an increase in IL-10-producing B cells and plasma cell infiltration in the gingiva with periodontitis, but the responsible B cell subtype has not been rigorously identified (118, 119). Consequently, the extent to which the decrease in peripheral blood B-1 cells reflects their involvement in tissue immunoregulation has not been fully explored.

On the other hand, analysis of B cells in nasal washings from children with mild and moderate bronchiolitis revealed a heterogeneous composition of the B cell pool, dominated by doubly negative (IgD^-^ CD27^-^) and naïve (IgD^+^ CD27^-^) B cells, with a lower presence of other subtypes, including B-1 cells (CD20^+^ CD27^+^ CD43^+^) (52). Interestingly, most of the B-1 cells in the nasal fluid were CD5^-^, unlike the proportions reported in peripheral blood (15, 48). Additionally, the repertoire analysis of Igs in the nasal fluid showed the presence of IgM with low SHM and skewed repertoire, suggesting a contribution of natural IgM in the control of viral infection; however, the cellular source of these antibodies was not studied (48). However, as this study did not measure B-1 cell proportions in healthy subjects or in the same children after infection, the effect of bronchiolitis on the B-1 cell pool and vice versa remains unknown.

Finally, it was shown that B-1 cells (CD20^+^ CD27^+^ CD43^+^) respond early to the pneumococcal vaccine, suggesting a greater capacity for antibody secretion in response to infection, as has been demonstrated in murine B-1 cells (35, 55, 120, 121).

Further studies are needed to determine whether B-1 cells are indeed associated with less severe outcomes during bacterial and viral infections, as well as the underlying mechanisms.

## Inflammatory and autoimmune diseases

### Cardiac diseases

B-1 cells have been studied in two immune-mediated cardiomyopathies: Chagas cardiomyopathy and dilated cardiomyopathy (DCM). In both conditions, B-1 cells appear to play an immunomodulatory role.

Chagas disease is caused by the intracellular parasite *Trypanosoma cruzi*. In the chronic phase of the disease, most patients remain asymptomatic (indeterminate form), and some develop life-threatening cardiomyopathy (122). Progression to Chagas cardiomyopathy is associated with the development of a chronic inflammatory response. However, several studies have shown that the infection induces the expansion of Breg cells, which may limit disease progression during the indeterminate form (123). Passos et al. (112), demonstrated that B-1 cells (CD20^+^ CD27^+^ CD43^+^) from patients with the indeterminate form of Chagas disease secrete IL-10 and TNF-α in response to a *T. cruzi*-derived protein, while B-1 cells from patients with chagasic heart disease do not. Furthermore, patients with the indeterminate form had more circulating CD11b^+^ B-1 cells, and co-culture with *T. cruzi*-derived protein amplified the frequency of this B-1 cell subpopulation. Moreover, the blood levels of CD11b^+^ B-1 cells from the same patients were positively correlated with improved cardiac function. Taken together, this suggests that B-1 cells, especially of the CD11b^+^ subtype, may be associated with cardioprotection in Chagas disease, although the mechanisms remain unknown.

DCM is a heart disease with diverse etiologies, characterized by structural and functional cardiac dysfunction. Increasing evidence demonstrates that the immune system plays a significant role in generating an environment of chronic inflammation and triggering autoimmune responses (124). In fact, the production of anticardiac antibodies, such as anti-β1-adrenergic (β1-AR) antibodies, can promote cardiac damage (125, 126). Recently, it was discovered that patients with idiopathic DCM have decreased levels of circulating B-1 cells (CD19^+^ CD20^+^ CD27^+^ CD43^+^ CD38^lo/int^), particularly those positive for anti-β1-AR antibodies. Furthermore, the percentage of circulating B-1 cells was positively correlated with preserved cardiac function (113). This evidence suggests that B-1 cells are not involved in the production of pathological autoantibodies in DCM or in the worsening of the disease, and could be protective.

### Multiple sclerosis

In multiple sclerosis (MS), several studies have pointed to B-1 cells (CD20^+^ CD43^+^ CD27^+^) as potential contributors, especially in the relapsing-remitting form, since these patients exhibit a reduced frequency of circulating B-1 cells, and their blood levels are inversely correlated with the time elapsed since the last relapse (114, 127). Interestingly, the frequency of circulating B-1 cells is not affected by medical treatment, except in the case of dimethyl fumarate (127, 128). However, further research is needed to determine whether B-1 cells play a significant role in MS.

### Systemic lupus erythematosus

Autoantibodies against nuclear antigens are known to contribute to the pathophysiology of SLE, particularly to the development of lupus nephritis (LN) (129). However, other autoantibodies have been proposed as contributors to kidney damage, including antiphospholipid antibodies like anti-PS (130, 131). In fact, anti-PS IgG levels are positively correlated with the development of LN and a higher disease activity index in both SLE patients and murine models of lupus. In mice, B-1a cells are the main producers of anti-PS IgG via the TLR/Syk signaling pathway (36). In fact, adoptive transfer of human anti-PS IgG and PS-specific murine B-1a cells accelerated kidney damage in mice. These results are consistent with findings in SLE patients, in whom circulating B-1 cells are the main source of anti-PS IgG. Furthermore, levels of kidney-infiltrating B-1 cells were positively correlated with proteinuria levels (36), suggesting that these cells contribute to the progression of LN.

It has also been proposed that CD11b^+^ B-1 cells are involved in SLE, due to their higher frequency in these patients, greater CD86 expression, and efficient ability to stimulate T-cell proliferation (56, 57). However, their contribution to the pathophysiology of SLE is less clear due to a lack of further studies comparing this subgroup of B-1 cells at different stages of the disease and with different complications.

### Other conditions

There are conditions in which the study of B-1 cells is even more scarce. For example, untreated inflammatory bowel disease patients have fewer circulating B cells subtypes, including fewer B-1 cells (defined by the authors as “CD20^+^CD27^+^ CD43^+^ pre-plasmablasts”) than patients treated with anti-TNFα antibodies (115). This finding shows that the therapy could modify the B cell levels in these patients but does not clarify the possible role of B-1 cells in inflammatory bowel disease. Similarly, another study showed that the percentage of B-1(CD20^+^ CD27^+^ CD43^lo–int^) and memory B cells is significantly reduced in patients with combined variable immunodeficiency (116), but the meaning of this finding remains unknown.

On the other hand, it is possible that human B-1 cells carry out tumor surveillance, since B-1 cells (CD20^+^ CD27^+^ CD43^+^ CD38^lo/int^) are the main producers of anti-Neu5GcGM3 IgM (33) which can eliminate tumor cells through complement-dependent and independent mechanisms (92). More studies are required to describe in detail the anti-tumor responses of B-1 cells in humans. Finally, another study demonstrated that B-1 cells (CD20^+^ CD27^+^ CD43^+^) react against blood group-A antigen and are enriched in ABO-incompatible kidney transplant patients (47). When anti-blood group A B cell subpopulations were analyzed in blood from healthy volunteers and peritoneal fluid from dialysis patients, B-1 cells showed the highest proportion of cells reactive to this antigen (47). However, this study did not confirm whether human B-1 cells could actually secrete these antibodies, so their contribution to the overall anti-blood group A antibody pool remains unknown.

## Concluding remarks

The main conclusion of this review is that B lymphocytes with functional characteristics like those of murine B-1 cells exist in humans. In fact, the CD combination CD19/CD20^+^ (or sIgM^+^), CD27^+^, CD43^+^ CD38^neg/low^ allows isolation of cells with features comparable to those of murine B-1 cells, such as tonic BCR signaling, spontaneous IgM secretion, enrichment of VH4–34 gene usage, and limited plasma cell differentiation. Furthermore, B-1 lymphocytes appear to contribute to the pool of the natural antibody-secreting cells in humans, with dsDNA and phosphorylcholine-specific antibodies being the best characterized.

**Figure 4:**
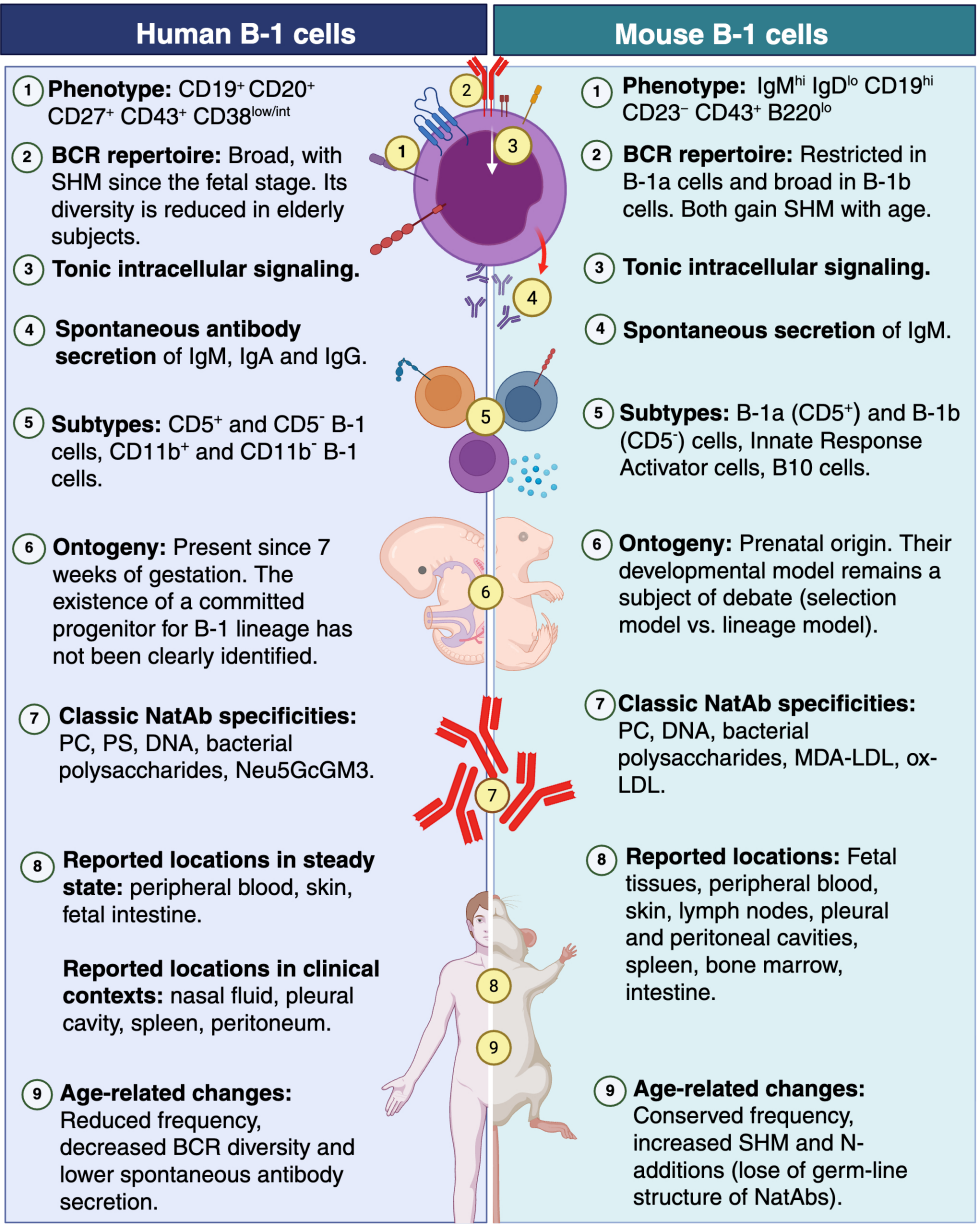
Integrative comparison of human and mouse B-1 cells. Abbreviations. SHM, somatic hypermutation; MDA-LDL, malondialdehyde-modified low-density lipoprotein; ox-LDL, Oxidized low-density lipoprotein cholesterol; NatAbs, natural antibodies; PC, phosphorylcholine; PS, phosphatidylserine Created in BioRender (License: BF29G2Y76U).

Human B-1 cells have unique characteristics (**Figure 4**). Unlike murine B-1 cells, human B-1 cells apparently originate from a progenitor shared with conventional B cells. However, early B-1 cells may still derive from non-hematopoietic stem cell (non-HSC) sources, as described in rodents, although this issue remains unresolved. The proportion of B-1 cells within the total B cell pool declines from birth to adulthood; however, no further decline is observed after 45–50 years of age, despite a continued reduction in the total number of B cells. Spontaneous antibody production, tonic BCR signaling, and repertoire diversity decrease in older individuals, likely due to clonal expansion and a shift in gene usage toward less autoreactive sequences.

### Unresolved issues and limitations

Despite advances in B-1 cell research, several controversies remain unresolved. The central debate is whether human B-1 cells constitute a distinct developmental lineage or represent a functional state within the pool of memory B cells. This question is complicated by single-cell transcriptomic data showing that CD27^+^ CD43^+^ cells are transcriptionally similar to activated memory B cells. The observation that these cells spontaneously secrete IgM without stimulation, exhibit a non-activated morphology, display a VH4-34–restricted repertoire with low diversity, and lack CD69 and CD70 expression supports the notion that they represent a distinct B cell subset. However, their ability to undergo class-switch recombination and respond in a T-dependent manner raises questions about whether they are functionally equivalent to murine B-1 cells, suggesting that “B-1” may represent convergent functional specialization rather than a conserved lineage.

Additionally, it remains unknown whether human B-1 cells migrate in response to inflammation or infection, and whether this migratory behavior could be related to the expression of integrins such as CD11b, as has been demonstrated in mice (9, 64). This uncertainty is linked to limitations imposed by restricted access to anatomical sites in humans, considered important niches for murine B-1 cells, such as body cavities.

Studies on human B-1 cells also face methodological limitations that continue to affect interpretation (e.g., inconsistent gating strategies, inadequate exclusion of doublets, or processing effects on CD43 and CD38 expression). Furthermore, across studies, there is limited functional comparison of several characteristics of human B-1 cells with their murine counterparts (e.g., affinity and polyreactivity of their NatAbs). Particularly in disease-related studies, the evidence is associative rather than causal, limiting the understanding of human B-1 cells during inflammation, infection, and autoimmune disorders.

On the other hand, this review has the limitation of not being a systematic review; therefore, although it is comprehensive, it may be subject to interpretative bias in the available literature, and relevant studies could have been overlooked. Additionally, because the scope of this review was restricted to murine and human B-1 cells, the conclusions exclude information from studies of B-1 or B-1-like cells in other species, such as non-human primates (132).

### Perspectives

We foresee that establishing a standardized reference CD panel in the near future is feasible and will facilitate further advancement of the field. Long-term progress will depend on integrating single-cell transcriptomic and epigenetic profiling with BCR repertoire analysis, together with functional tests directly comparing human B-1 cells, memory B cells, and plasmablasts under identical conditions. Ultimately, determining whether human B-1 cells represent a distinct developmental lineage or a conserved functional subset will require harmonized definitions and consistent multi-omic approaches across laboratories.
