# Supplementary material for: Do human B-1 lymphocytes truly exist?
Source: Front Immunol. 2026 Apr 13;17:1810144. doi: 10.3389/fimmu.2026.1810144 (PMC13111098; doi:10.3389/fimmu.2026.1810144)
Supplement: Supplementary file 1 [file Table1.docx]

**Supplementary material**

**Supplementary Table 1**. Definitions for human “B-1” and “B-1-like” cell phenotypes

| **Term** | **Phenotype** | **Notes** | **References** |
| --- | --- | --- | --- |
| **Human B-1 cell** | - CD19⁺/CD20⁺ CD27⁺ CD43⁺ CD38^low/int^ - Alternatives:   - CD19⁺/CD20⁺ CD27⁺ CD43⁺   - sIgM^+^ CD27^+^ CD43^+^ | Complete phenotype  This population has very few activated B cells (CD69^+^/CD70^+^ or CD38^hi^)  Surface IgM substitutes CD20 (used in fetal tissues) | (1)  (1,2)  (3) |
| **Human “B-1-like” cells** | - CD19^+^CD43^+^ CD38^-^ CD5^+^ - CD19⁺/CD20⁺ CD5^+^ | Incorrect or partial phenotypes | (4–7) |

**Supplementary Table 2**. Characteristics of mouse B-1a and B-1b cells

| **Feature** | **B-1 a cells** | **B-1 b cells** | **Reference** |
| --- | --- | --- | --- |
| Surface marker | IgM^hi^ IgD^lo^ CD19^hi^ CD23^−^ CD43^+^ B220^lo^ **CD5^+^** | IgM^hi^ IgD^lo^ CD19^hi^ CD23^−^ CD43^+^ B220^lo^ **CD5^-^** | (8,9) |
| Origin | Prenatal precursors, with some input from the adult bone marrow. | Prenatal precursors, with relevant contribution of adult bone marrow. | (10–12) |
| Frequency | Body cavities as the main reservoir (35-70% of B cells) | Do not represent the major subtype in many B-1 cell niches. | (9) |
| NatAb secretion | Main producers of natural Abs, critical in homeostasis maintenance and early infection control. In this sense, B-1a cells are the most studied and their hallmark is the secretion of anti-PC NatAbs expressing the T15 idiotype. | | (13–18) |
| Atherosclerosis protection | Secretion of atheroprotective anti-MDA-LDL, ox-LDL and CuOx-LDL IgM. Their frequency is associated with reduced plaque burden. B-1b cells produce more of these antibodies *in vivo* than B-1a cells. | | (18,19) |
| Class switch capacity | IgM as the main isotype. Class-switch to IgG and IgA to a lesser extent than B-1b cells. | Class-switch to IgA and to a lesser extent IgG3. | (19–21) |
| Contribution to gut IgA | Share prenatal origin with IgA plasma cells, but they do not substantially contribute to the adult IgA plasma cell pool. | Important source (after B-2 cells) of commensal-specific IgA plasma cells in the adult intestine. | (10,22) |
| Repertoire | Restricted, with use of germline V(D)J segments. Progressively gain SHM and N-additions with age. | Broad, since the neonatal period. Gain N-additions with age. | (14,23–26) |
| Response to infection | Migration, IgM and GM-CSF secretion. Only a fraction of B-1a cells differentiate into plasma cells. | Provide long-lasting protection (T-cell–independent memory B cells). | (13,20,27–34) |
| Differentiation capacity | Plasma cells, IL-10 secreting cells, innate response activator (IRA) cells, phagocyte-like cells. | Phagocyte and macrophage-like cells. | (13,35–38) |
| Age-related changes in NatAbs | Natural anti-PC IgM loses its germ-line structure by gaining N-additions, resulting in lower protection against *S. pneumoniae* (except in aged female mice). | B-1b cells are increased in aged hyperlipidemic mice. Possess greater diversity and IgM secretion than B-1a cells. Predominant responders to pneumococcal vaccine. | (26,39–43) |
| Role in autoimmune diseases | Dual role. Are involved in lupus pathophysiology and in protective functions in the case of other autoimmune diseases. | Dual role. They produce Abs related to transplantation rejection (anti-Gal IgM). At the same time, their anti-GlcNAc Abs seem to delay autoimmune diabetes development. | (44–53) |

*Abbreviations: Abs: antibodies. MDA: malondialdehyde. LDL: low density lipoprotein. CuOx-LDL: LDL oxidized by copper. Gal: Galα1-3Galβ1-4GlcNAc. GlcNAc: N-acetyl-D-glucosamine. PC: phosphorylcholine. NatAb: natural antibody. SHM: somatic hypermutation.*

**Supplementary table 3.** Studies of “B-1-like” cells defined by CD19^+^ CD5^+^ phenotype in various diseases

| **Condition** | **Findings** | **Reference** |
| --- | --- | --- |
| Septic peritonitis | Increased in the peritoneal fluid of patients.  Positive correlation with disease severity. | (5) |
| Peritoneal dialysis and bilateral tube ligation (BTL). | In BTL patients CD19^+^ CD5^+^ cells represented 63% of B cells and in the peritoneal dialysis group these cells represented 38% of B cells. | (4) |
| Multiple sclerosis (MS) | A high percentage of blood CD5^+^ B cells is a risk factor for earlier conversion of clinical isolated syndromes to MS. | (6) |
| Septic Shock | The levels of CD19^+^ CD5^+^ B cells are decreased in patients with septic shock, although these changes do not increase the mortality risk. | (7) |

**References**

1. Quách TD, Rodríguez-Zhurbenko N, Hopkins TJ, Guo X, Hernández AM, Li W, Rothstein TL. Distinctions among Circulating Antibody-Secreting Cell Populations, Including B-1 Cells, in Human Adult Peripheral Blood. *J Immunol* (2016) 196:1060–1069. doi: 10.4049/jimmunol.1501843

2. Griffin DO, Holodick NE, Rothstein TL. Human B1 cells in umbilical cord and adult peripheral blood express the novel phenotype CD20+ CD27+ CD43+ CD70-. *J Exp Med* (2011) 208:67–80. doi: 10.1084/jem.20101499

3. Suo C, Dann E, Goh I, Jardine L, Kleshchevnikov V, Park J-E, Botting RA, Stephenson E, Engelbert J, Tuong ZK, et al. Mapping the developing human immune system across organs. *Science* (2022) 376:eabo0510. doi: 10.1126/science.abo0510

4. Donze HH, Lue C, Julian BA, Kutteh WH, Kantele A, Mestecky J. Human peritoneal B-1 cells and the influence of continuous ambulatory peritoneal dialysis on peritoneal and peripheral blood mononuclear cell (PBMC) composition and immunoglobulin levels. *Clin Exp Immunol* (1997) 109:356–361. doi: 10.1046/j.1365-2249.1997.4541352.x

5. von Loeffelholz C, Winkler R, Weigel C, Piskor E-M, Vivas W, Rauchfuß F, Settmacher U, Rubio I, Weis S, Gräler MH, et al. Increased peritoneal B1-like cells during acute phase of human septic peritonitis. *iScience* (2024) 27:110133. doi: 10.1016/j.isci.2024.110133

6. Villar LM, Espiño M, Roldán E, Marín N, Costa-Frossard L, Muriel A, Alvarez-Cermeño JC. Increased peripheral blood CD5+ B cells predict earlier conversion to MS in high-risk clinically isolated syndromes. *Mult Scler Houndmills Basingstoke Engl* (2011) 17:690–694. doi: 10.1177/1352458510396922

7. Monserrat J, de Pablo R, Diaz-Martín D, Rodríguez-Zapata M, de la Hera A, Prieto A, Alvarez-Mon M. Early alterations of B cells in patients with septic shock. *Crit Care* (2013) 17:R105. doi: 10.1186/cc12750

8. Prieto JMB, Felippe MJB. Development, phenotype, and function of non-conventional B cells. *Comp Immunol Microbiol Infect Dis* (2017) 54:38–44. doi: 10.1016/j.cimid.2017.08.002

9. Yenson V, Baumgarth N. “Purification and Immune Phenotyping of B-1 Cells from Body Cavities of Mice.,” In: Vitale G, Mion F, editors. *Regulatory B Cells: Methods and Protocols*. New York, NY: Springer (2014). p. 17–34 doi: 10.1007/978-1-4939-1161-5_2

10. Vergani S, Muleta KG, Silva CD, Doyle A, Kristiansen TA, Sodini S, Krausse N, Montano G, Kotarsky K, Nakawesi J, et al. A self-sustaining layer of early-life-origin B cells drives steady-state IgA responses in the adult gut. *Immunity* (2022) 55:1829-1842.e6. doi: 10.1016/j.immuni.2022.08.018

11. Ghosn EEB, Yamamoto R, Hamanaka S, Yang Y, Herzenberg LA, Nakauchi H, Herzenberg LA. Distinct B-cell lineage commitment distinguishes adult bone marrow hematopoietic stem cells. *Proc Natl Acad Sci U S A* (2012) 109:5394–5398. doi: 10.1073/pnas.1121632109

12. Montecino-Rodriguez E, Leathers H, Dorshkind K. Identification of a B-1 B cell–specified progenitor. *Nat Immunol* (2006) 7:293–301. doi: 10.1038/ni1301

13. Aziz M, Holodick NE, Rothstein TL, Wang P. The role of B-1 cells in inflammation. *Immunol Res* (2015) 63:153–166. doi: 10.1007/s12026-015-8708-3

14. Prohaska TA, Que X, Diehl CJ, Hendrikx S, Chang MW, Jepsen K, Glass CK, Benner C, Witztum JL. Massively parallel sequencing of peritoneal and splenic B cell repertoires highlights unique properties of B-1 cell antibodies. *J Immunol Baltim Md 1950* (2018) 200:1702–1717. doi: 10.4049/jimmunol.1700568

15. Baumgarth N, Tung JW, Herzenberg LA. Inherent specificities in natural antibodies: a key to immune defense against pathogen invasion. *Springer Semin Immunopathol* (2005) 26:347–362. doi: 10.1007/s00281-004-0182-2

16. Choi YS, Baumgarth N. Dual role for B-1a cells in immunity to influenza virus infection. *J Exp Med* (2008) 205:3053–3064. doi: 10.1084/jem.20080979

17. Baumgarth N, Herman OC, Jager GC, Brown L, Herzenberg LA, Herzenberg LA. Innate and acquired humoral immunities to influenza virus are mediated by distinct arms of the immune system. *Proc Natl Acad Sci* (1999) 96:2250–2255. doi: 10.1073/pnas.96.5.2250

18. Chou M-Y, Fogelstrand L, Hartvigsen K, Hansen LF, Woelkers D, Shaw PX, Choi J, Perkmann T, Bäckhed F, Miller YI, et al. Oxidation-specific epitopes are dominant targets of innate natural antibodies in mice and humans. *J Clin Invest* (2009) 119:1335–1349. doi: 10.1172/JCI36800

19. Rosenfeld SM, Perry HM, Gonen A, Prohaska TA, Srikakulapu P, Grewal S, Das D, McSkimming C, Taylor AM, Tsimikas S, et al. B-1b Cells Secrete Atheroprotective IgM and Attenuate Atherosclerosis. *Circ Res* (2015) 117:e28–e39. doi: 10.1161/CIRCRESAHA.117.306044

20. Baumgarth N. The double life of a B-1 cell: self-reactivity selects for protective effector functions. *Nat Rev Immunol* (2011) 11:34–46. doi: 10.1038/nri2901

21. Roy B, Shukla S, Łyszkiewicz M, Krey M, Viegas N, Düber S, Weiss S. Somatic hypermutation in peritoneal B1b cells. *Mol Immunol* (2009) 46:1613–1619. doi: 10.1016/j.molimm.2009.02.026

22. Bunker JJ, Flynn TM, Koval JC, Shaw DG, Meisel M, McDonald BD, Ishizuka IE, Dent AL, Wilson PC, Jabri B, et al. Innate and adaptive humoral responses coat distinct commensal bacteria with immunoglobulin A. *Immunity* (2015) 43:541–553. doi: 10.1016/j.immuni.2015.08.007

23. Yang Y, Wang C, Yang Q, Kantor AB, Chu H, Ghosn EE, Qin G, Mazmanian SK, Han J, Herzenberg LA. Distinct mechanisms define murine B cell lineage immunoglobulin heavy chain (IgH) repertoires. *eLife* (2015) 4:e09083. doi: 10.7554/eLife.09083

24. Holodick NE, Repetny K, Zhong X, Rothstein TL. Adult BM generates CD5+ B1 cells containing abundant N-region additions. *Eur J Immunol* (2009) 39:2383–2394. doi: 10.1002/eji.200838920

25. Tornberg UC, Holmberg D. B‐1a, B‐1b and B‐2 B cells display unique VHDJH repertoires formed at different stages of ontogeny and under different selection pressures. *EMBO J* (1995) 14:1680–1689. doi: 10.1002/j.1460-2075.1995.tb07157.x

26. Srikakulapu P, Pattarabanjird T, Upadhye A, Bontha SV, Osinski V, Marshall MA, Garmey J, Deroissart J, Prohaska TA, Witztum JL, et al. B-1b Cells Have Unique Functional Traits Compared to B-1a Cells at Homeostasis and in Aged Hyperlipidemic Mice With Atherosclerosis. *Front Immunol* (2022) 13: doi: 10.3389/fimmu.2022.909475

27. Smith FL, Baumgarth N. B-1 Cell Responses to Infections. *Curr Opin Immunol* (2019) 57:23–31. doi: 10.1016/j.coi.2018.12.001

28. Hiéronimus L, Huaux F. B-1 cells in immunotoxicology: Mechanisms underlying their response to chemicals and particles. *Front Toxicol* (2023) 5:960861. doi: 10.3389/ftox.2023.960861

29. Baumgarth N, Waffarn EE, Nguyen TTT. Natural and induced B-1 cell immunity to infections raises questions of nature versus nurture. *Ann N Y Acad Sci* (2015) 1362:188–199. doi: 10.1111/nyas.12804

30. Yang Y, Tung JW, Ghosn EEB, Herzenberg LA, Herzenberg LA. Division and differentiation of natural antibody-producing cells in mouse spleen. *Proc Natl Acad Sci U S A* (2007) 104:4542–4546. doi: 10.1073/pnas.0700001104

31. Waffarn EE, Hastey CJ, Dixit N, Soo Choi Y, Cherry S, Kalinke U, Simon SI, Baumgarth N. Infection-induced type I interferons activate CD11b on B-1 cells for subsequent lymph node accumulation. *Nat Commun* (2015) 6:8991. doi: 10.1038/ncomms9991

32. Alugupalli KR, Leong JM, Woodland RT, Muramatsu M, Honjo T, Gerstein RM. B1b lymphocytes confer T cell-independent long-lasting immunity. *Immunity* (2004) 21:379–390. doi: 10.1016/j.immuni.2004.06.019

33. Haas KM. Programmed Cell Death 1 Suppresses B-1b Cell Expansion and Long-Lived IgG Production in Response to T Cell-Independent Type 2 Antigens. *J Immunol* (2011) 187:5183–5195. doi: https://doi.org/10.4049/jimmunol.1101990

34. Ordoñez C, Savage HP, Tarajia M, Rivera R, Weeks‐Galindo C, Sambrano D, Riley L, Fernandez PL, Baumgarth N, Goodridge A. Both B‐1a and B‐1b cells exposed to Mycobacterium tuberculosis lipids differentiate into IgM antibody‐secreting cells. *Immunology* (2018) 154:613–623. doi: 10.1111/imm.12909

35. Chousterman BG, Swirski FK. Innate response activator B cells: origins and functions. *Int Immunol* (2015) 27:537–541. doi: 10.1093/intimm/dxv028

36. Gao J, Ma X, Gu W, Fu M, An J, Xing Y, Gao T, Li W, Liu Y. Novel functions of murine B1 cells: Active phagocytic and microbicidal abilities. *Eur J Immunol* (2012) 42:982–992. doi: 10.1002/eji.201141519

37. Popi AF, Motta FLT, Mortara RA, Schenkman S, Lopes JD, Mariano M. Co-ordinated expression of lymphoid and myeloid specific transcription factors during B-1b cell differentiation into mononuclear phagocytes in vitro. *Immunology* (2009) 126:114–122. doi: 10.1111/j.1365-2567.2008.02883.x

38. Almeida SR, Aroeira LS, Frymuller E, Dias MÂA, Bogsan CSB, Lopes JD, Mariano M. Mouse B-1 cell-derived mononuclear phagocyte, a novel cellular component of acute non-specific inflammatory exudate. *Int Immunol* (2001) 13:1193–1201. doi: 10.1093/intimm/13.9.1193

39. Haas KM. B-1 lymphocytes in mice and non-human primates. *Ann N Y Acad Sci* (2015) 1362:98–109. doi: 10.1111/nyas.12760

40. Quách TD, Hopkins TJ, Holodick NE, Vuyyuru R, Manser T, Bayer R-L, Rothstein TL. Human B-1 And B-2 B Cells Develop From Lin−CD34+CD38lo Stem Cells. *J Immunol Baltim Md 1950* (2016) 197:3950–3958. doi: 10.4049/jimmunol.1600630

41. Benedict CL, Kearney JF. Increased junctional diversity in fetal B cells results in a loss of protective anti-phosphorylcholine antibodies in adult mice. *Immunity* (1999) 10:607–617. doi: 10.1016/s1074-7613(00)80060-6

42. Webster SE, Ryali B, Clemente MJ, Tsuji NL, Holodick NE. Sex Influences Age-Related Changes in Natural Antibodies and CD5+ B-1 Cells. *J Immunol Baltim Md 1950* (2022) 208:1755–1771. doi: 10.4049/jimmunol.2101150

43. Webster SE, Tsuji NL, Clemente MJ, Holodick NE. Age-related changes in antigen-specific natural antibodies are influenced by sex. *Front Immunol* (2023) 13: doi: 10.3389/fimmu.2022.1047297

44. Ma K, Du W, Wang S, Xiao F, Li J, Tian J, Xing Y, Kong X, Rui K, Qin R, et al. B1-cell-produced anti-phosphatidylserine antibodies contribute to lupus nephritis development via TLR-mediated Syk activation. *Cell Mol Immunol* (2023) 20:881–894. doi: 10.1038/s41423-023-01049-2

45. Ohdan H, Swenson KG, Kruger Gray HS, Yang Y-G, Xu Y, Thall AD, Sykes M. Mac-1-Negative B-1b Phenotype of Natural Antibody-Producing Cells, Including Those Responding to Galα1,3Gal Epitopes in α1,3-Galactosyltransferase-Deficient Mice1. *J Immunol* (2000) 165:5518–5529. doi: 10.4049/jimmunol.165.10.5518

46. She Z, Li C, Wu F, Mao J, Xie M, Hun M, Abdirahman AS, Luo S, Wan W, Tian J, et al. The Role of B1 Cells in Systemic Lupus Erythematosus. *Front Immunol* (2022) 13: doi: 10.3389/fimmu.2022.814857

47. Zhong X, Lau S, Bai C, Degauque N, Holodick NE, Steven SJ, Tumang J, Gao W, Rothstein TL. A Novel Subpopulation of B-1 Cells Is Enriched With Autoreactivity in Normal and Lupus-Prone Mice. *Arthritis Rheum* (2009) 60:3734–3743. doi: 10.1002/art.25015

48. Enghard P, Humrich JY, Chu VT, Grussie E, Hiepe F, Burmester G-R, Radbruch A, Berek C, Riemekasten G. Class switching and consecutive loss of dsDNA-reactive B1a B cells from the peritoneal cavity during murine lupus development. *Eur J Immunol* (2010) 40:1809–1818. doi: 10.1002/eji.200940050

49. Xu Z, Morel L. Contribution of B-1a cells to systemic lupus erythematosus in the NZM2410 mouse model. *Ann N Y Acad Sci* (2015) 1362:215–223. doi: 10.1111/nyas.12607

50. Takahashi T, Strober S. Natural killer T cells and innate immune B cells from lupus-prone NZB/W mice interact to generate IgM and IgG autoantibodies. *Eur J Immunol* (2008) 38:156–165. doi: 10.1002/eji.200737656

51. Ito T, Ishikawa S, Sato T, Akadegawa K, Yurino H, Kitabatake M, Hontsu S, Ezaki T, Kimura H, Matsushima K. Defective B1 cell homing to the peritoneal cavity and preferential recruitment of B1 cells in the target organs in a murine model for systemic lupus erythematosus. *J Immunol Baltim Md 1950* (2004) 172:3628–3634. doi: 10.4049/jimmunol.172.6.3628

52. Choi JK, Yu C-R, Bing SJ, Jittayasothorn Y, Mattapallil MJ, Kang M, Park SB, Lee H-S, Dong L, Shi G, et al. IL-27–producing B-1a cells suppress neuroinflammation and CNS autoimmune diseases. *Proc Natl Acad Sci* (2021) 118:e2109548118. doi: 10.1073/pnas.2109548118

53. New JS, Dizon BLP, King RG, Greenspan NeilS, Kearney JF. B-1 B cell-derived natural antibodies against N-acetyl-D-glucosamine suppress autoimmune diabetes pathogenesis. *J Immunol Baltim Md 1950* (2023) 211:1320–1331. doi: 10.4049/jimmunol.2300264
